# Whole-Genome Sequence of *Corynebacterium pseudotuberculosis* PA04, Isolated from the Lymph Node of a Sheep in the Amazon, Brazil

**DOI:** 10.1128/genomeA.00202-17

**Published:** 2017-04-20

**Authors:** Wana L. O. Costa, Jorianne T. C. Alves, Larissa M. Dias, Carlos Leonardo de Aragão Araújo, Eziquiel Morais, André G. M. Silva, Soraya S. Andrade, Rommel T. J. Ramos, Artur Silva, Adriana R. C. Folador

**Affiliations:** a Center of Genomics and System Biology, Laboratory of Genomic and Bioinformatics, Federal University of Pará, Belem, Pará, Brazil; b Federal University of Pará, Campus of Castanhal, Pará, Brazil

## Abstract

This study reports the complete genome sequence of *Corynebacterium pseudotuberculosis* strain PA04, isolated from a sheep in the Amazon, Brazil. This bacterium is the etiological agent of caseous lymphadenitis. This genome contains 2,338,093 bp, 52.2% G+C content, and a total of 2,104 coding sequences (CDSs), 41 pseudogenes, 12 rRNAs, and 49 tRNAs.

## GENOME ANNOUNCEMENT

*Corynebacterium pseudotuberculosis* is a Gram-positive bacterium which is the causative agent of caseous lymphadenitis (CLA), a chronic disease that affects small ruminants, mostly sheep and goats, although it also affects other animals, such as horses, cattle, buffalo, and, in rare cases, humans (1–3).

*C. pseudotuberculosis* infections are found worldwide, with higher prevalence in meat-producing countries, such as Australia, New Zealand, South Africa, the United States, Canada, and Brazil (1, 2, 4). In Brazil, most of the reported cases are from the Northeast (5). In the northern part of Brazil, according to the report of the Brazilian Institute of Geography and Statistics (IBGE) in 2015 (http://www.ibge.gov.br/estadosat/temas.php?siglapa&temapecuaria2015), most sheep and goat herds in the region are from the State of Pará, which contribute 50% of the goat and 36% of the sheep herd breeding in the north of Brazil. However, the lack of data related to cases of CLA underestimates the actual prevalence of this disease in the state (6). This absence of information may be explained by deficiencies in notification of this disease in many countries (7).

Whole-genome sequencing of *C. pseudotuberculosis* has contributed to the use of omics approaches to study particular characteristics of this pathogen, its ability to cause infection, and its gene expression (8), and to predict valuable proteins for drug target investigations (9, 10).

*C. pseudotuberculosis* strain PA04 is the third complete genome to be published from the State of Pará. It was isolated from a mandibular lymph node puncture from a male Dorper breed sheep in Pará, northern Brazil. This strain has been deposited in a Brazilian collection.

Identification was performed by biochemical and molecular methods, using the API Coryne kit (bioMérieux, USA) and PCR multiplex with *rpoB*, 16S, and *pld* genes, respectively (11). The genome was sequenced by the Ion Torrent PGM platform using the 318 Chip, with a fragment library, where a total of 560,337,368 bp were produced, with a genomic coverage of 239×. The quality of the raw data was evaluated using FastQC (http://www.bioinformatics.babraham.ac.uk/projects/fastqc/), and filtering and trimming were performed using the FASTX-Toolkit (http://hannonlab.cshl.edu/fastx_toolkit/) to remove reads with Phred quality scores below 20. The genome assembly was performed using the Mira software (12), which provided 40 contigs with an *N*_*50*_ of 221,388 bp and a total size of 2,353,386 bp. The number of contigs was reduced to four using the SeqMan Pro tool of the Lasergene 11 Core Suite (DNAStar), and the scaffold was generated by Mauve (13). Gap closure was performed by CLC Genomics Workbench (CLC bio).

This genome was automatically annotated using Rapid Annotations using Subsystems Technology (RAST) 2.0 (14). The RNAmmer 1.2 software (15) was used for the prediction of tRNAs and rRNAs. The coding sequence (CDS) correction was performed using the Artemis software (16) associated with BLASTp (https://blast.ncbi.nlm.nih.gov/Blast.cgi?PAGE=Proteins) and UniProt (http://www.uniprot.org) databases.

This genome contains 2,338,093 bp, with a G+C content of 52.2% and a total of 2,104 CDSs, 41 pseudogenes, 12 rRNAs, and 49 tRNAs.

### Accession number(s).

This whole-genome project has been deposited at the GenBank database under the accession number CP019587.
